# The Impact of Nutritional Management on Fat-Soluble Nutrient Status in Patients with Fatty Acid Oxidation Disorders: A Cross-Sectional Study

**DOI:** 10.3390/metabo16020124

**Published:** 2026-02-11

**Authors:** Maria Wasiewicz-Gajdzis, Małgorzata Jamka, Łukasz Kałużny, Natalia Wichłacz-Trojanowska, Anna Blask-Osipa, Monika Duś-Żuchowska, Joanna Jagłowska, Szymon Kurek, Anna Miśkiewicz-Chotnicka, Jarosław Walkowiak

**Affiliations:** 1 Poznan University of Medical Sciences, Department of Pediatric Gastroenterology and Metabolic Diseases, Szpitalna 27/33, 60-572 Poznan, Poland; maria.wasiewiczgajdzis@ump.edu.pl (M.W.-G.); mjamka@ump.edu.pl (M.J.); lkaluzny@ump.edu.pl (Ł.K.); natalia.wichlacztrojanowska@ump.edu.pl (N.W.-T.); mduszuchowska@ump.edu.pl (M.D.-Ż.); skurek@ump.edu.pl (S.K.); chotnicka@ump.edu.pl (A.M.-C.); 2 Department of Pediatrics, Hematology and Oncology, Medical University of Gdansk, Dębinki Str. 7, 80-211 Gdansk, Poland

**Keywords:** fat-modified diet, inborn errors of metabolism, nutrition, retinol, cholecalciferol, α-tocopherol, β-carotene

## Abstract

**Background:** Fatty acid oxidation disorders (FAOD) are rare inborn errors of metabolism that impair mitochondrial β-oxidation and energy production. Management includes fasting avoidance for all FAOD types. Patients with long-chain FAOD are advised to restrict long-chain triglycerides (LCTs) to 10% of total energy intake and supplement medium-chain triglycerides (MCTs). The impact of such dietary modification on fat-soluble vitamin status has not yet been studied. **Methods:** In this cross-sectional study, serum concentrations of vitamins A, 25(OH)D, E, and β-carotene were measured in 36 FAOD patients and 36 healthy controls matched for age and sex. Vitamins A, E, and β-carotene were quantified using high-performance liquid chromatography and vitamin 25(OH)D through an immunoassay. FAOD patients were further divided into fat-modified (LCT-restricted) and standard-fat diet subgroups based on dietary management. **Results:** FAOD patients had significantly higher vitamin A concentrations than controls (*p* < 0.05), while there was no difference in vitamins 25(OH)D, E, and β-carotene. Within the FAOD cohort, the fat-modified group had higher levels of vitamins A and 25(OH)D but lower levels of vitamin E and β-carotene than the standard-fat group (all *p* < 0.05). Vitamin 25(OH)D deficiency (<20 ng/mL) was more frequent in the standard-fat group (*p* = 0.03). **Conclusions:** Fat-modified diets influence fat-soluble vitamin status in FAOD, emphasising the importance of ongoing monitoring and tailored supplementation. Future work should focus on optimising nutritional management, including modifications to formula composition, and on addressing the currently limited evidence on nutritional status and vitamin deficiencies in patients with FAOD.

## 1. Introduction

Fatty acid oxidation is a complex mitochondrial pathway that converts fatty acids released from adipose tissue into acetyl-CoA and reducing equivalents, which are utilised for energy production, especially under conditions of limited glucose availability, during fasting, exercise, or febrile illness [1,2]. Fatty acids are also a preferred energy source for the myocardium in the neonatal period, for skeletal muscle during sub-maximal exercise, and for the liver [1,3]. Mutations in genes encoding fatty acid transport proteins and enzymes involved in fatty acid oxidation cause fatty acid oxidation disorders (FAOD), which are inherited in an autosomal recessive manner [1]. FAODs result in defective energy metabolism and accumulation of toxic acylocarnitines [1,2,4,5]. There are at least 20 known FAODs, with a combined incidence of 0.9–15.2 per 100,000, with medium-chain acyl-CoA dehydrogenase deficiency (MCAD) being the most common (1 per 20,000 in Northern European/Caucasian populations) [6,7]. Among long-chain fatty acid oxidation disorders (LC-FAOD), very long-chain acyl-CoA dehydrogenase deficiency (VLCAD) has the highest incidence (1 per 30,000 to 100,000 births) [8,9], followed by long-chain 3-hydroxyacyl-CoA dehydrogenase deficiency (LCHAD)—1 per 50,000 to 170,000 births [7,10]. The incidence of FAODs is believed to be increasing due to the wide implementation of newborn screening programmes (NBS) [7,11,12].

The clinical presentation of FAOD varies depending on the type of defect, but some common features can be identified. Because fatty acid oxidation is an important energy source for organs with high metabolic demands, clinical symptoms predominantly affect the liver, heart, skeletal muscle, and central nervous system [9,13,14,15,16]. During acute episodes of metabolic stress, the patient may present with hypoglycemia without ketones, elevation of transaminases, and elevated ammonia [5]. Progression of symptoms to hepatic insufficiency, brain oedema, and death is possible. Additionally, LC-FAODs, such as LCHAD and VLCAD, as well as certain diseases of carnitine metabolism, including carnitine acylcarnitine translocase deficiency (CACT) and carnitine palmitoyltransferase I deficiency (CPT1), may present with cardiomyopathy and rhabdomyolysis. Neuropathy and retinopathy occur exclusively in patients with LCHAD [1,5,17].

Management depends on the type of FAOD. In cases of LC-FAODs, fasting should be avoided, and dietary management typically includes restricting long-chain triglycerides (LCTs) to approximately 10% of total energy intake [18,19] while supplementing with medium-chain triglycerides (MCT oil or MCT-enhanced formula) at about 20% of total energy intake [9]. A newer treatment option for LC-FAOD is triheptanoin. This synthetic medium-chain odd-chain triglyceride was approved for patient use in the United States in 2020 but is only available through compassionate programmes in other countries [14,20]. Patients with MCAD and short-chain acyl-CoA dehydrogenase deficiency (SCAD) are advised to avoid fasting [21], and dietary fat restrictions are not recommended [9]. Patients with carnitine uptake defect (CUD) require carnitine supplementation and fasting avoidance [12].

The dietary modifications required in FAOD may place patients at risk of nutritional disturbances. It is well established that exclusion or modified diets used to treat certain diseases predispose patients to micro- and macronutrient deficiencies and disturbances in nutritional state [22,23,24]. Patients with LC-FAOD, who must follow significant restrictions in fat intake, are unable to consume many natural sources of fat-soluble nutrients such as plant oils, nuts, fish, full-fat dairy, and eggs [25]. Poor intake of nutrients, including vitamins K and E and essential fatty acids (linoleic and linolenic acid), has previously been documented in children with LCHAD [18]. Some guidelines [26,27] and reviews [28] highlight the possibility of deficiencies in fat-soluble micronutrients; however, robust scientific evidence is lacking.

We hypothesised that FAOD patients are more likely to experience disturbances in the status of vitamins A, D, E, and β-carotene. Therefore, we aimed to assess fat-soluble vitamin concentrations in FAOD patients compared with healthy subjects and to examine the differences within the FAOD group according to dietary management strategy (fat-modified diet versus standard-fat diet).

## 2. Materials and Methods

### 2.1. Ethical Approval

This cross-sectional study [29] was conducted between 2020 and 2025. The study protocol was approved by the Poznań University of Medical Sciences Ethical Committee (approval no. 803/20), and all procedures were performed in accordance with the Helsinki Declaration of 1975 [30].

### 2.2. Study Population

The FAOD patients were recruited at the Department of Pediatric Gastroenterology and Metabolic Diseases at the University of Medical Sciences in Poznań, Greater Poland Voivodeship, Poland, between November 2020 and February 2025. Blood acylcarnitine assays, urinary organic acid profiles, and genetic testing confirmed the diagnosis of FAOD in all patients [16,31,32]. Patients born after December 2013 were diagnosed through NBS [33]. Inclusion criteria included age (1–20 years) and willingness to participate. Exclusion criteria included current metabolic decompensation, acute illness, or pregnancy. Due to the exploratory nature of the study, all candidates who expressed willingness to participate were enrolled.

Sex- and age-matched control subjects (aged 1 to 20 years) were recruited through a family medicine practice clinic in Wronki, Greater Poland Voivodeship, Poland, between September 2024 and February 2025. Exclusion criteria included the presence of acute or chronic disease and mental or physical disability. Information regarding the use of vitamins and dietary supplements was obtained from all participants. Written and oral consent was obtained from all study participants and their legal guardians.

### 2.3. Subgroup Analysis

To assess whether there were differences between FAOD patients treated with a diet restricted in LCTs and those receiving a standard-fat diet, the patients were divided into two subgroups:

- Fat-modified diet group—patients treated with LCTs restriction: 9 patients with LCHAD and 5 patients with VLCAD.
- Standard-fat diet group—patients managed with fasting avoidance (16 patients with MCAD and 1 patient with SCAD) and carnitine (5 patients with CUD).

### 2.4. Anthropometric Measurements

Measurements were taken in the morning by study physicians, in light clothing and without shoes, using Seca 703 scales (Hamburg, Germany) for participants > 2 years of age and Seca 376 scales for participants < 2 years of age. Weight was measured with an accuracy of 0.1 kg and height with an accuracy of 0.1 cm. Based on these measurements, BMI was calculated with a precision of 0.1 kg/m^2^.

To determine the percentiles of body weight, height, and BMI in study participants under 18, WHO growth charts were used for children < 3 years of age [34] and OLA/OLAF growth charts for children aged 3–18 [35].

### 2.5. Blood Sampling

Venous blood was drawn after a fasting period of 10 h from the vein of the antecubital fossa. The length of fasting time did not exceed the recommended safe periods for FAOD patients [26,27,36,37]. The blood samples were protected from light with aluminum foil, centrifuged, aliquoted, and stored at −80 °C until analysis.

### 2.6. Biochemical Analysis

Vitamins A, E, and β-carotene were measured at the Laboratory of the Department of Pediatric Gastroenterology and Metabolic Diseases using high-performance liquid chromatography (HPLC; Hewlett-Packard 1100 Series HPLC System, Wladbronn, Germany) as previously described [38,39]. The reference ranges were 300–750 ng/mL for vitamin A [40,41], 5.7–19.920 µg/mL for vitamin E [42], and 30–910 µg/mL for β-carotene [43,44]. Vitamin 25(OH)D concentrations were measured in a commercial laboratory (Diagnostyka S.A., Poznan, Poland) using an immunoassay on the Alinity i analyser (Abbott, Chicago, IL, USA). Vitamin 25(OH)D status was assessed per the Polish Guidelines for Preventing and Treating vitamin D Deficiency, according to which 25(OH)D < 20 ng/mL indicates deficiency; 20–30 ng/mL suboptimal status; 30–50 ng/mL adequate to optimal; 50–100 ng/mL high supply; and >100 ng/mL increased intoxication risk [45]

### 2.7. Data Quality and Bias Control

We applied several strategies to control for bias, including a prospective study design, clear and consistent inclusion and exclusion criteria, standardised measurement protocols and validated instruments operated by experienced, well-trained staff, predefined outcomes and endpoints, a pre-specified statistical analysis plan, and reporting in accordance with the STROBE guidelines. There were no missing data, as all eligible FAOD patients who attended our metabolic centre during the study period were consecutively enrolled. All assessed markers were available for both patients and healthy controls. Population-based controls were recruited from the same geographic area as the FAOD group (Greater Poland Voivodeship). Furthermore, propensity score matching for age and sex was used to select the control group.

### 2.8. Sample Size Calculation

Sample size estimation was performed using G*Power 3.1 (Heinrich Heine University Düsseldorf, Düsseldorf, Germany). The calculation was based on anticipated differences in serum vitamin 25(OH)D concentrations between the study groups and assumed a two-sided significance level of 0.05, a statistical power of 80%, a mean difference of 20% between groups, a standard deviation estimated at 30% of the mean, and equal allocation of participants to each group (1:1). Based on these assumptions, a minimum of 30 participants per group were required. To account for a potential dropout rate of up to 10%, the target recruitment was increased to 34 participants per group to maintain sufficient statistical power.

### 2.9. Statistical Analysis

Continuous data are presented as means (M) with standard deviations (SD), medians (Me), and interquartile ranges (Q1–Q3). Categorical variables are presented as absolute and relative frequencies.

Controls were selected using propensity score matching, adjusting for age and sex. The Shapiro–Wilk test was used to assess the normality of the parameters. When the parameters followed a Gaussian distribution, the equality of variances was tested using Levene’s test. If the assumption of equal variances was met, Student’s *t*-test was used to test for differences between the two groups; otherwise, Welch’s *t*-test was used. When a non-normal distribution of data was observed, the Mann–Whitney U test with continuity correction was used to compare the groups. A Chi^2^ test was used to check for differences in sex distribution. *p* < 0.05 was considered statistically significant.

To analyse the associations between categorical variables, the likelihood-ratio Chi^2^, Pearson’s Chi^2^, and Fisher’s exact test (two-sided) were used. Statistica Software v.14 (TIBCO Software Inc., Palo Alto, CA, USA) was used for all calculations.

## 3. Results

### 3.1. Clinical Characteristics

**Figure 1:**
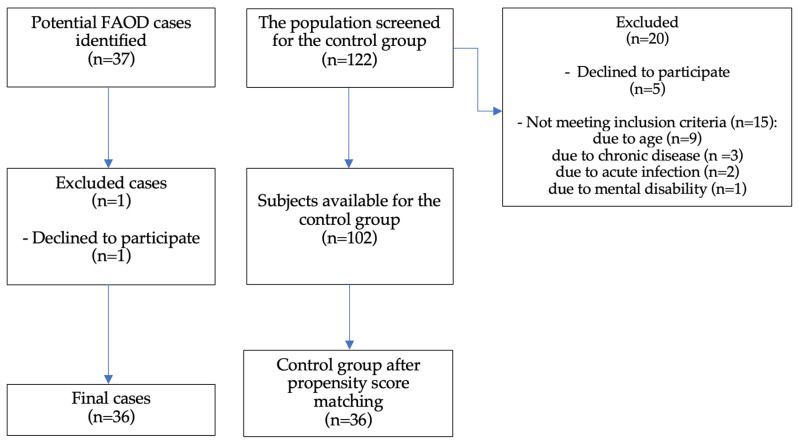
Sample selection flowchart. FAOD—fatty acid oxidation disorders.

The sample selection flowchart (Figure 1) outlines the recruitment process. We have included 36 out of 37 FAOD patients (one patient declined to participate). A total of 122 individuals were initially screened for inclusion in the control group. Of these, 20 were excluded: 5 declined to participate, and 15 did not meet the inclusion criteria. The reasons for exclusion due to lack of eligibility included age (*n* = 9), chronic disease (*n* = 3), acute infection (*n* = 2), and mental disability (*n* = 1). As a result, 102 participants were available for the control group. Propensity score matching was subsequently performed, resulting in a final matched control group of 36 participants. Table 1 summarises population characteristics. The FAOD group consisted of 16 patients with MCAD (44%), 9 with LCHAD (25%), 5 with VLCAD (14%), 5 with CUD (14%), and 1 with SCAD (3%). Four patients (12%) were born before the introduction of NBS in Poland. There were no statistically significant differences in any of the recorded anthropometric parameters between the FAOD group and the control group.

### 3.2. Vitamin Concentrations—FAOD vs. Control Group

The findings are summarised in Table 1. No significant differences in serum concentrations of vitamin 25(OH)D, vitamin E, or β-carotene were observed between the FAOD group and the control group. The FAOD group exhibited a significantly higher vitamin A concentration.

At the individual level, 11.1% of the FAOD group had vitamin 25(OH)D deficiency (<20 ng/mL), compared with 5.6% of controls. Suboptimal vitamin 25(OH)D levels (20–30 ng/mL) were observed in 30.6% and 36.1% of subjects, respectively. No participant had a potentially toxic vitamin 25(OH)D concentration (>100 ng/mL). Vitamin A levels were below the reference range in 8.3% of the FAOD group compared with 19.4% of controls; one control participant (2.8%) exceeded 750 ng/mL. β-carotene concentrations were below the reference range in 22.2% of the FAOD group and 5.6% of controls. All but one FAOD patient (2.7%) had vitamin E concentrations within the reference range. Overall, no statistically significant differences in the prevalence of excessive, normal, or deficient levels of vitamin A (*p* = 0.18), D (*p* = 0.65), E (*p* = 1.00), and β-carotene (*p* = 0.08) were observed between FAOD patients and controls.

### 3.3. Vitamin Concentrations—Fat-Modified Group vs. Standard-Fat Group

The comparison of vitamin concentrations between the fat-modified group and the standard-fat group is presented in Table 2. There were no statistically significant differences in sex structure or anthropometric parameters between the groups. Statistically significant differences in the serum concentrations of all fat-soluble vitamins were observed. Vitamin 25(OH)D and vitamin A concentrations were higher in the fat-modified group. In contrast, the concentrations of vitamin E and β-carotene were significantly higher in the standard-fat diet group.

No patients on a fat-restricted diet had vitamin 25(OH)D deficiency (<20 ng/mL) compared with 18.2% on a non-fat-restricted diet. Suboptimal 25(OH)D levels (20–30 ng/mL) were observed in 14.3% vs. 40.9% subjects, respectively. No participant in either group exhibited a toxic level of vitamin 25(OH)D; however, 13.6% of patients in the non-fat-restricted group had vitamin A deficiency, and the remaining subjects with FAOD had normal vitamin A levels. β-carotene deficiency was common, 36.7% in the fat-restricted group and 13.6% in the non-fat-restricted group. Vitamin E values were normal in all but one FAOD patient (7.14%). In summary, vitamin 25(OH)D deficiency was statistically more prevalent in the standard-fat diet group (*p* = 0.03). No statistically significant differences were found between the fat-modified group and the standard-fat group in the prevalence of excessive or deficient levels of vitamins A (*p* = 0.27), E (*p* = 0.39) and β-carotene (*p* = 1.0).

## 4. Discussion

Our study investigated the serum concentrations of fat-soluble vitamins in patients with FOAD and compared them to those of healthy control subjects. While no statistically significant differences were observed in the concentrations of vitamin 25(OH)D, E, and β-carotene, the FAOD group exhibited significantly higher vitamin A levels.

Interestingly, patients on the fat-modified diet had significantly higher concentrations of vitamins A and 25(OH)D but lower concentrations of vitamin E and β-carotene than those on the standard-fat diet. We observed a higher percentage of deficient and suboptimal vitamin 25(OH)D levels in the standard-fat diet group compared with the fat-restricted diet group (59.1% vs. 14.3%). There was also a high percentage of decreased β-carotene concentrations in the fat-modified group compared to the standard-fat diet group (50% vs. 22.7%, respectively). In the standard fat group, three patients (13.6%) had decreased vitamin A concentrations; in both groups, vitamin A and E were within reference ranges.

To date, micronutrient deficiencies in FAOD have been poorly studied in the scientific literature. One study by Gillingham et al. [18] revealed deficient intake of fat-soluble vitamins E and K, assessed by a three-day diet record. VLCAD treatment guidelines recommend assessing fat-soluble vitamin levels in patients due to the risk of deficiency [27].

The higher concentrations of vitamins A and 25(OH)D observed in the fat-modified diet group are likely due to the regular use of formulas (Table 3) and supplements. Fortified medical foods (such as Modulen or Lipistart) contain substantial amounts of vitamins A and D. Supplements used by some patients, particularly fish liver oil, are a natural source of both vitamins [46,47]. In the fat-modified group, 9 (64.3%) patients used formula, including 4 (28.6%) who additionally supplemented with vitamin D and 3 (21.4%) who used fish oil preparations. One (7.1%) patient took multiple vitamin preparation and one (7.1%) a vitamin D supplement. Considering vitamin use, 10 (71.4%) patients received supplementary doses of vitamin A, D, and E, and 1 (7.1%) subject received vitamin D. In the standard-fat diet group, 12 (54.5%) patients were supplemented with vitamin D preparations and 2 (9.1%) with fish oil. Considering vitamin use, 2 patients (9.1%) received vitamins A, D, and E, whereas 12 patients (54.5%) received only vitamin D. In the control group, 21 (61.8%) subjects were supplemented with vitamin D. The frequency of vitamin D supplementation was not significantly higher in the fat-modified group (78.5% vs. 63.6% vs. 61.8%). However, considering formula intake, the amount consumed from additional sources was definitely higher. Vitamin A and E supplementary intake was significantly more frequent in the fat-modified group than in the standard-fat diet (71.4% vs. 16.7%, *p* = 0.0002).

Several factors may have contributed to lower vitamin E concentrations in the fat-modified group compared to the standard-fat group. Firstly, patients are restricted to provide only 10% of their total energy intake from LCTs [36], which limits their ability to consume foods naturally rich in vitamin E, such as vegetable oils and nuts. The bioavailability of tocopherol is enhanced by the fat content of the meal [48,49]. Consistently, tocopherol acetate (a vitamin E precursor) showed greater bioaccessibility in long-chain than in medium-chain triglyceride emulsions [50]. Given that patients in the fat-modified group use 100% MCT oil for cooking, the intake and absorption of vitamin E may be reduced. Furthermore, the bioavailability of vitamin E is influenced by the food matrix of the meal. Research has demonstrated that vitamin E bioaccessibility varies depending on the type of produce, being very low in apples but almost complete in bananas, bread, or lettuce [51], and differing between pasta types depending on the egg content [52]. Similarly, Cilla et al. [53] reported greater α-tocopherol bioaccessibility in cow milk-based fruit beverages than in soy-based drinks. A study by Jeanes et al. [48] found that both the amount of fat and the meal matrix significantly influenced α-tocopherol absorption.

Lower concentrations of β-carotene in the fat-modified diet group could be explained by both decreased bioavailability from meals and lack of supplementation. Medical foods consumed by patients on the fat-modified diet do not contain β-carotene. Furthermore, the bioaccessibility of β-carotene increases significantly (from 14% to 86%) with increasing LCT fraction (from 0% to 100%) [54]. It was documented that the bioaccessibility of β-carotene from carrots [55] and peppers [56] was significantly higher when co-digested with emulsions containing long-chain fatty acids than with emulsions based on medium- or short-chain fatty acids. Therefore, as in the case of vitamin E, the absorption of β-carotene may be affected negatively by the LCT restriction [57]. The food matrix and its processing influence β-carotene bioavailability. Disruption of plant cell walls enhances carotenoid release and micellarization, thereby improving bioavailability. In vitro studies have shown that the homogenization and cooking of carrots increased β-carotene accessibility from 3% in raw samples to 27–39% when cooked and combined with oil [58]. Furthermore, the ingestion of carrot juice caused a two times the increase in plasma β-carotene compared with raw carrots [59]. In addition, the patients did not receive β-carotene supplementation.

Our study’s strengths include a robust sample size (compared with other case-control studies of patients with IEM) and a well-matched control group. The limitations of this study include the lack of a detailed, long-term quantitative assessment of dietary intake and supplementation, which could be correlated with serum vitamin concentrations. The evaluation of meal patterns and preparation techniques that affect the bioaccessibility of fat-soluble nutrients in patients with FOAD would allow for a more robust analysis. Interpretation of the results is hampered by the lack of widely accepted reference ranges and by the limited number of studies establishing normative vitamin A and β-carotene concentrations in healthy children. Increasing the sample size of LC-FAOD patients could improve subgroup analysis.

## 5. Conclusions

Generally, FAOD patients have higher vitamin A concentrations than healthy controls. Interestingly, fat-modified diets were associated with higher vitamin A and 25(OH)D concentrations and lower vitamin E and β-carotene levels compared to a standard-fat diet, reflecting the combined impact of supplementation, food fortification, and reduced bioavailability under LCT restriction. These findings emphasise the importance of tailored nutritional and supplementation monitoring in FAOD and warrant larger studies with detailed dietary assessments. In particular, future work should focus on optimising nutritional management, including modifications to formula composition, and on addressing the currently limited evidence on nutritional status and vitamin deficiencies in patients with FAOD.

## Figures and Tables

**Table 1:** Characteristics of the study population.

	FAOD *n* = 36	Control *n* = 36	*p*
**Age (years)**			0.84 *
Mean ± SD	7.9 ± 4.5	8.1 ± 4.6	
Median	7.2	7.8	
Q1–Q3	(4.6–11.6)	(4.6–11.7)	
**Sex (*n* (%))**			0.81 ^#^
Females	20 (55.6%)	18 (50.0%)	
Males	16 (44.4%)	18 (50.0%)	
**Weight (kg)**			0.95 ^#^
Mean ± SD	35.0 ± 22.1	33.3 ± 18.6	
Median	25.1	27.1	
Q1–Q3	(18.8–52.8)	(18.8–44.5)	
**Height (cm)**			0.87 *
Mean ± SD	129.1 ± 29.5	130.2 ± 30.2	
Median	123.0	126.0	
Q1–Q3	(113.0–152.0)	(107.0–155.5)	
**BMI (kg/m^2^)**			0.86 ^#^
Mean ± SD	18.5 ± 4.6	17.9 ± 3.0	
median	18.0	17.8	
Q1–Q3	(14.7–21.5)	(16.0–19.7)	
**Weight Percentile**			0.65 ^#^
Median	77.5	61.2	
Q1–Q3	(33.0–93.5)	(37.0–88.0)	
**Height Percentile**			0.21 ^#^
Median	75.5	60	
Q1–Q3	(53.5–92.0)	(26.0–95.0)	
**BMI Percentile**			0.60 ^#^
Median	68.0	69.0	
Q1–Q3	(20.0–91.0)	(40.0–88.0)	
**Vitamin 25(OH)D** (**ng/mL**)			0.87 ^#^
Mean ± SD	33.98 ± 15.76	33.41 ± 9.84	
Median	33.00	32.20	
Q1–Q3	(22.45–40.15)	(26.60–42.25)	
**Vitamin A (Retinol, ng/mL)**			**0.03** ^#^
Mean ± SD	426.21 ± 99.87	390.97 ± 113.88	
Median	430.27	364.08	
Q1–Q3	(365.29–474.75)	(320.50–420.50)	
**Vitamin E (Tocopherol, µg/mL)**			0.25 *
Mean ± SD	8.40 ± 1.69	8.84 ± 1.55	
Median	8.24	9.05	
Q1–Q3	(7.17–9.43)	(7.70–10.00)	
**β-carotene** (**ng/mL**)			0.58 ^#^
Mean ± SD	102.53 ± 107.74	96.50 ± 62.31	
Median	84.68	74.50	
Q1–Q3	(36.21–11.76)	(53.65–131.40)	

BMI—body mass index, FAOD—fatty acid oxidation disorders, SD—standard deviation, Q1–Q3—interquartile range; * Student *t*-Test; ^#^ U Mann–Whitney Test.

**Table 2:** Comparison of the fat-modified diet group and the standard-fat group.

	Fat-Modified Diet Group *n* = 14	Standard-Fat Diet Group *n* = 22	*p*
**Age (years)**			0.65 *
Mean ± SD	8.3 ± 5.3	7.6 ± 4.1	
Median	7.3	6.9	
Q1–Q3	(4.7–11.6)	(4.6–11.6)	
**Sex (*n* (%))**			0.85 ^#^
Females	7 (50%)	13 (59%	
Males	7 (50%)	9 (41%)	
**Weight (kg)**			0.76 ^#^
Mean ± SD	37.7 ± 26.8	33.3 ± 19.0	
Median	24.0	26.5	
Q1–Q3	(21.0–54.0)	(17.0–51.6)	
**Height (cm)**			0.69 *
Mean ± SD	131.6 ± 32.4	127.6 ± 28.2	
Median	126.2	123.0	
Q1–Q3	(112.0–152.0)	(114.0–152.0)	
**BMI (kg/m^2^)**			0.90 ^#^
Mean ± SD	18.7 ± 5.3	18.4 ± 4.2	
median	16.3	18.4	
Q1–Q3	(14.6–22.1)	(14.8–21.2)	
**Weight Percentile**			0.45 ^#^
Median	81.5	73.0	
Q1–Q3	(34–96)	(32.0–92.0)	
**Height Percentile**			0.56 ^#^
Median	79.5	67.8	
Q1–Q3	(57.0–92.0)	(49.0–92.0)	
**BMI Percentile**			0.72 ^#^
Median	63.5	68.0	
Q1–Q3	(22.0–94.0)	(20.0–90.0)	
**Vitamin 25(OH)D (ng/mL)**			**0.02** ^#^
mean ± SD	41.19 ± 16.41	29.40 ± 13.81	
median	35.7	27.35	
Q1–Q3	(32.60–43.60)	(21.50–35.70)	
**Vitamin A (Retinol, ng/mL)**			**0.03** *
Mean ± SD	470.02 ± 74.43	398.33 ± 105.35	
Median	471.56	398.92	
Q1–Q3	(430.18–528.21)	(341.40–444.22)	
**Vitamin E (Tocopherol, µg/mL)**			**0.01** ^#^
Mean ± SD	7.49 ± 1.78	8.98 ± 1.37	
Median	7.14	9.02	
Q1–Q3	(6.30–8.09)	(8.09–9.61)	
**β-carotene** (**ng/mL**)			**0.04** ^#^
Mean ± SD	59.17 ± 40.95	130.13 ± 127.50	
Median	54.78	99.68	
Q1–Q3	(22.88–104.18)	(50.90–147.29)	

BMI—body mass index, FAOD—fatty acid oxidation disorders, SD—standard deviation, Q1–Q3—interquartile range; * Student *t*-test; ^#^ U Mann–Whitney Test.

**Table 3:** Vitamin contents of several types of MCT-based products.

	Monogen (Nutricia)		Lipistart (Vitaflo)		MCTprocal (Vitaflo)	MCTOil (Nutricia)
	per 100 g	per 100 mL	per 100 g	per 100 mL	per 100 g	per 100 mL
Vitamin A (µg)	325	54.6	466	69	0	0
Vitamin D_3_ (µg)	11.9	2.0	9.2	1.8	0	0
Vitamin E (mg)	4.9	0.82	11	1.5	0	0
β-carotene	0 *	0 *	0 *	0 *	0	0

* Information not provided on the nutritional label.

## Data Availability

The original contributions presented in this study are included in the article. Further inquiries can be directed to the corresponding author.

## Abbreviations

BMI	body mass index
CACT	carnitine–acylcarnitine translocase
CPT1	carnitine palmitoyltransferase I deficiency
CUD	carnitine uptake defect
25(OH)D	vitamin 25(OH)D
FAOD	fatty acid oxidation disorders
HPLC	high-performance liquid chromatography
LCHAD	long-chain 3-hydroxyacyl-CoA dehydrogenase deficiency
LCT	long-chain triglycerides
MCAD	medium-chain acyl-CoA dehydrogenase
MCT	medium-chain triglycerides
Me	median
NBS	newborn screening
OLA/OLAF	growth charts for the Polish population aged 3–18
Q1–Q3	quartile 1–quartile 3
SCAD	short-chain acyl-CoA dehydrogenase deficiency
SD	standard deviation
VLCAD	very long-chain acyl-CoA dehydrogenase deficiency
WHO	World Health Organization
